# Online Questionnaire with Fibromyalgia Patients Shows Negative Correlations between Disease Severity and Adherence to Mediterranean Diet

**DOI:** 10.3390/nu16071078

**Published:** 2024-04-06

**Authors:** Elisa Proietti, Fabio Rapallo, Elena Molinari, Viviana Mucci, Lucio Marinelli, Consuelo Borgarelli, Bruno Burlando, Livia Pisciotta, Ilaria Demori

**Affiliations:** 1 Department of Internal Medicine (DIMI), University of Genova, Viale Benedetto XV, 6, 16132 Genova, Italy; proietti.elisa93@gmail.com (E.P.); consuelo.borgarelli@unige.it (C.B.); livia.pisciotta@unige.it (L.P.); 2 Department of Economics (DIEC), University of Genova, Via Vivaldi, 5, 16126 Genova, Italy; fabio.rapallo@unige.it; 3 Clincal Psychology Center, Division of Neurology, E.O. Ospedali Galliera, Via Mura delle Cappuccine 14, 16128 Genova, Italy; elena.molinari@galliera.it; 4 School of Science, Western Sydney University, Penrith, NSW 2750, Australia; v.mucci@westernsydney.edu.au; 5 Department of Neuroscience, Rehabilitation, Ophthalmology, Genetics, Maternal and Child Health (DINOGMI), University of Genova, Largo P. Daneo 3, 16132 Genova, Italy; lucio.marinelli@unige.it; 6 IRCCS Ospedale Policlinico San Martino, Department of Neuroscience, Division of Clinical Neurophysiology, Largo R. Benzi 10, 16132 Genova, Italy; 7 Department of Pharmacy (DIFAR), University of Genova, Viale Benedetto XV 3, 16132 Genova, Italy; bruno.pietro.burlando@unige.it; 8 IRCCS Ospedale Policlinico San Martino, Department of Internal Medicine, Operative Unit of Dietetics and Clinical Nutrition, Largo R. Benzi 10, 16132 Genova, Italy

**Keywords:** pain, food habits, lifestyle, dietary supplements, body mass index

## Abstract

Fibromyalgia (FM) is a multidimensional disorder in which intense chronic pain is accompanied by a variety of psychophysical symptoms that impose a burden on the patients’ quality of life. Despite the efforts and the recent advancement in research, FM pathogenesis and effective treatment remain unknown. Recently, the possible role of dietary patterns and/or components has been gaining attention. The current study aimed to investigate a potential correlation between adherence to the Mediterranean diet (MedDiet) and FM severity in a sample of Italian FM patients. An online survey was designed, composed of customized questions and validated questionnaires with the aim of investigating the intensity and type of pain, the presence of other psychophysical symptoms, the overall impact of FM, general food and lifestyle habits, and adherence to the MedDiet. The collected responses were analyzed for descriptive statistics, linear regression, and propensity score analyses. The results show that, despite considerable use of pharmaceuticals and supplements, FM participants suffered from a high-severity grade disease. However, those with good adherence to the MedDiet experienced a lower pain intensity and overall FM impact. A propensity score analysis indicates a positive influence of the MedDiet against FM severity, thus unveiling the need for well-designed intervention studies to evaluate the therapeutic potential of different dietary patterns.

## 1. Introduction

Fibromyalgia (FM) is a multifaceted, invalidating syndrome mainly characterized by multisite chronic pain accompanied by fatigue, sleep problems, and brain fog [1,2]. It has a prevalence of about 1–5% in the adult population, with a worldwide average female-to-male ratio of 3:1 [3]. Accumulated evidence suggests that FM is a central disorder characterized by nociplastic pain generated by altered processing of non-painful or mildly painful stimuli [4,5]. However, knowledge of FM pathophysiology is scarce and correlated to the lack of biological or clinical biomarkers, making the diagnosis of FM challenging and delayed by several years [6]. Diagnostic criteria are based on the persistence of pain spreading in all body quadrants for more than 3 months and on the presence of pain caused by digital pressure in a minimum of 11 out of 18 specific tender points [7,8,9]. In addition to this, patients are known to report a long list of other symptoms and comorbidities, such as anxiety, depression, headaches, dizziness, joint stiffness, paresthesia, dysautonomia, and gastrointestinal problems. Such a complicated clinical landscape hinders the definition of therapeutic guidelines, thus making patient management and prognosis complex and difficult [5,10]. FM is usually treated with a multimodal approach, with variable outcomes and no resolutive solution [8,11,12]. The most frequently adopted drugs in pharmacological treatments include amitriptyline, duloxetine or milnacipran, tramadol, pregabalin, and cyclobenzaprine [13]. The most common non-pharmacological and alternative treatments include physical therapy and exercise, massages, relaxation, psychotherapy, acupuncture, and dietary interventions [6].

Diet is a major factor influencing the microbiota–gut–brain axis, and dysbiosis has been linked to multiple central disorders, including altered nociception [14]. The prevalence of obesity, dysbiosis, and gastrointestinal disorders in FM [3] suggests a role of nutrition and the microbiota–gut–brain axis in the pathogenesis of the disease. Although these relationships have not yet been elucidated, evidence is growing on the role of specific dietary components in modulating FM symptoms [15]. The deficiency in some minerals and vitamins (e.g., magnesium, selenium, vitamin D, and vitamin B12), as well as the inadequate intakes of anti-oxidant and anti-inflammatory compounds and the increased consumption of dietary excitotoxins, have been associated with the worsening of FM pain and psychophysical manifestations [16,17]. 

Intervention studies assessing the effects exerted by specific dietary patterns on FM patients are still insufficient [18,19]. The Mediterranean diet (MedDiet) has been recognized by the UNESCO Cultural Heritage of Humanity since 2010 (United Nations Educational, Scientific and Cultural Organization. The Mediterranean Diet. Intangible Heritage and UNESCO 2010, https://ich.unesco.org/en/decisions/5.COM/6.41, accessed on 12 January 2024). The MedDiet pattern stands out from other dietary approaches due to the synergistic combination of high-quality foods rich in vitamins; minerals; fiber; anti-inflammatory and anti-oxidant nutrients, such as polyphenols, carotenoids, monounsaturated fatty acids (MUFA); and polyunsaturated fatty acids (PUFA) from the *n*-3 series [20,21]. Several rigorous studies established the MedDiet benefits in the prevention and treatment of various diseases including cardiovascular diseases, metabolic imbalance, and cancer [22,23,24]. 

Given these premises, we conducted a questionnaire-based study designed to describe clinical traits, lifestyle, eating habits, and the use of pharmacological and non-pharmacological therapies in a sample consisting of *n* = 186 Italian FM patients. The primary aim was to investigate a potential correlation between adherence to the MedDiet and the severity state of FM, intended as the impact of the disease on daily life, the intensity and type of pain, and psychological well-being.

## 2. Materials and Methods

### 2.1. Ethical Approval and Patient Recruitment

Prior to patient recruitment, this study was approved by the Research Ethics Committee of the University of Genova (Assent N. 2023/51). Then, all investigations were conducted according to the principles expressed in the Declaration of Helsinki. An online anonymous survey was opened from June to September 2023, accessible through a link on the Microsoft Office365 Platform of the University of Genova (Microsoft Forms^®^, Microsoft, Redmond, WA, USA). Participants were recruited by an Italian association of people affected by FM (Fibromialgia Comitato Assoutenti Liguria), which shared with 280 addresses from its mailing list an information letter and the link to the survey. Participants were asked to check off inclusion/exclusion criteria to participate in the survey. Inclusion criteria were being over 18 years of age and having an official and established FM diagnosis (symptoms having been present for at least 3 months) [5]. Exclusion criteria were unfamiliarity with the Italian language, pregnancy or breastfeeding, abuse of substances or alcohol, and psychotic disorders. Participants joined the survey individually, freely, and anonymously. The acceptance of full informed consent was mandatory to be able to start the survey.

This is an observational descriptive study without the need to recruit a healthy comparison group [25]. The comparison is made between the MedDiet adherence score and FM severity state.

### 2.2. Questionnaires

The first section of the survey on FM patients assessed socio-demographic characteristics such as age, sex, education, marital status, employment, and, in addition, clinical aspects, including biometrics and disease duration. Symptoms other than pain were recorded on a presence/absence basis, and patients were asked to indicate any medications they used against their syndrome. Customized questions were also inserted to investigate the patients’ lifestyles and eating habits. 

For pain evaluation, participants reported their average global pain intensity over the past 24 h on an 11-point Numerical Rating Scale (NRS) ranging from 0 = “no pain” to 10 = “worst imaginable pain”. A more detailed analysis of pain was realized through the painDETECT questionnaire (PD-Q), which investigates pain types and characteristics. The PD-Q was developed to detect neuropathic/nociplastic pain components, especially in chronic patients [26]. A “low” score (≤12) indicates a prevalent nociceptive pain component, a “high” score (≥19) indicates that a neuropathic or central pain component is >90% likely, and an “intermediate” condition is considered in between. 

For the evaluation of overall psychological well-being, participants answered the Depression, Anxiety, and Stress Scale—21 Items (DASS-21), which is composed of 3 self-assessment scales [27]. FM patients scored specific statements based on their frequency or intensity on a 4-point Likert scale (ranging from 1 = “never” to 4 = “always”). The respondents are categorized according to the presence and severity of symptoms of depression, anxiety, and stress (normal, mild, moderate, severe, extremely severe), as defined by specific cut-off points. 

The severity state of FM was assessed through the Revised Fibromyalgia Questionnaire (FIQR) [28]. The FIQR is composed of questions designed to collect information on three domains of FM patients’ quality of life: physical function (FIQR1, 9 items), overall impact (FIQR2, 2 items), and symptoms (FIQR3, 10 items). The questions cover a variety of aspects, including pain level, fatigue, stiffness, physical function, and ability to carry out daily activities over the last week. Patients respond to the various items on a scale from 0 to 10 so that FIQR1 maximum value is 90, FIQR2 maximum value is 20, and FIQR3 maximum value is 100. To obtain the total FIQR score, the domain 1 score is divided by 3, the domain 2 score remains unchanged, and the domain 3 score is divided by 2. By adding the 3 resulting values, the total FIQR score reaches, at most, the value of 100. A categorization of FM severity grade is made according to cut-off points: remission, ≤30; mild, between 31 and 45; moderate, between 46 and 65; and severe, >65 [29].

Finally, to evaluate the adherence of FM participants to the MedDiet, we used the PREDIMED-14, a reformulated and reduced questionnaire based on the PREDIMED study. PREDIMED-14 consists of 14 questions relating to the habitual frequency of consumption, or amount consumed, of 12 main components of the MedDiet (including extra virgin olive oil, fruit and vegetables, fish or seafood, legumes, meat, and sweets) and two food habits related to the MedDiet [30]. Each item is scored 1 or 0, depending on whether participants adhere or not to each diet component; thus, the resulting score ranges from 0 to 14. Values ≤ 5 indicate poor adherence to the MedDiet, whilst a score ≥ 10 indicates good adherence; an intermediate condition is considered in between.

### 2.3. Statistical Analyses

Sociodemographic and clinical data were analyzed by descriptive statistics, including mean, median, quartiles, and standard deviation for continuous variables, and percentages for categorical variables. Graphical summaries included empirical cumulative distribution functions and boxplots for univariate distributions and scatterplots for bivariate distributions. 

To visually assess the relationship between two variables in the scatterplot, we used the LOESS (LOcally Estimated Scatterplot Smoothing) regression with degree 2. Correlation analyses were conducted using linear regression. A binary dummy variable was created for the PREDIMED-14 (1 in case of good adherence; 0 otherwise), and a regression with the dummy predictor was considered. To confirm the results with a causal analysis, we considered the same regression using a propensity score technique, with inverse probability weighting based on suitable possible confounders. Significance of the regression was assessed with the F-test. 

All data analyses were carried out using the software R (version 4.2.2, https://www.r-project.org/, accessed on 9 October 2023).

## 3. Results

### 3.1. Demographics and Clinical Characteristics

**Figure 1:**
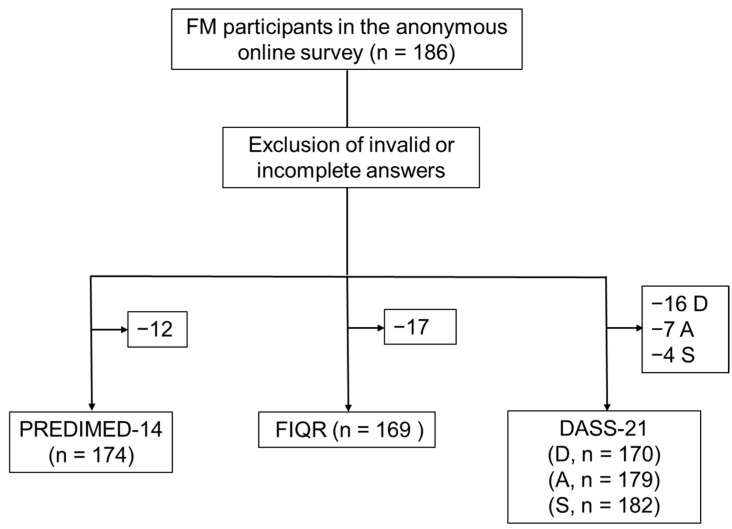
Workflow of invalid and incomplete answers exclusion and data analysis. FIQR: Revised Fibromyalgia Questionnaire; DASS-21: Depression (D), Anxiety (A), and Stress (S) Scale-21 Items.

The survey received 186 answers from FM participants, accounting for 66% of the total number of invitations sent. The exclusion of invalid or incomplete answers is explained in the flow diagram of Figure 1.

FM patients were prevalently females with upper secondary or higher education (74%), were married or cohabitant, and with grey-collar employment or unemployed (Table 1). Most participants (88%) were diagnosed by a rheumatologist, while only 3.3% were diagnosed by a neurologist, and the remaining ones were diagnosed by other medical specialists.

As shown in Table 2, the median participants’ age was 56 years, and the patients had been suffering from FM for 11 years on average. The reported height and weight information was used to calculate the participants’ body mass index (BMI, kg/m^2^). The median BMI of 25.6 indicated the prevalence of overweight (BMI > 24.9) in 50% of the subjects, which was markedly higher than the prevalence observed in the Italian adult population (33%). The prevalence of obesity (BMI > 30) in the study sample was also higher than the overall prevalence in Italy (25% vs. 10%) [31].

As Table 3 shows, the participants were administered complex pharmacological therapies, combining anti-inflammatories, analgesics, antidepressants, antineuralgics, and anxiolytics (Table 3a). Supplements were also widely used, particularly vitamin D, magnesium, and prebiotics/probiotics (Table 3b). Only about 14% of the participants stated they did not take any of the above. 

The majority of the participants (57.5%) also chose non-pharmacological mind–body therapies, such as physiotherapy and meditation techniques (Table 4).

**Figure 2:**
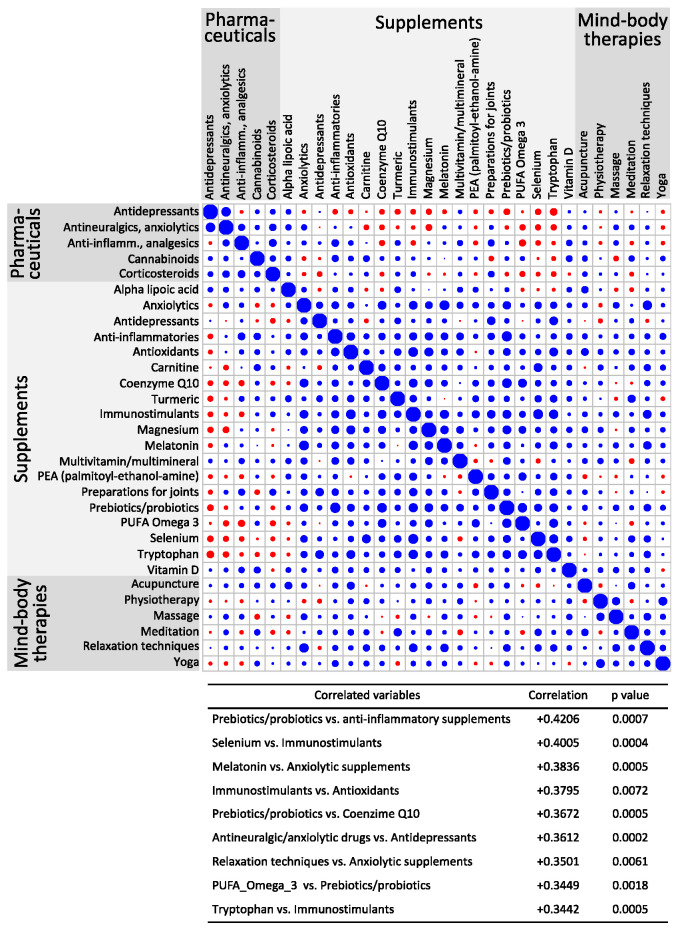
Matrix of the correlations among dummy variables representing YES/NO answers about the use of pharmaceuticals, supplements, and non-pharmacological mind–body therapies. Each dot represents the correlation between the variables of the corresponding row and column, the size indicates the strength, and the color of the sign implies the following: blue, positive; red, negative. Strongest correlations and their *p* values are numerically detailed in the bottom panel.

A matrix of pairwise correlations among the use of pharmaceuticals, supplements, and non-pharmacological mind–body therapies is reported in Figure 2. A general tendency to combine therapeutic approaches of the same type can be observed. For example, the strongest positive correlation was found between anti-inflammatory supplements and prebiotics/probiotics, followed by the association of natural anxiolytics with melatonin.

### 3.2. FM Severity and Symptoms

**Figure 3:**
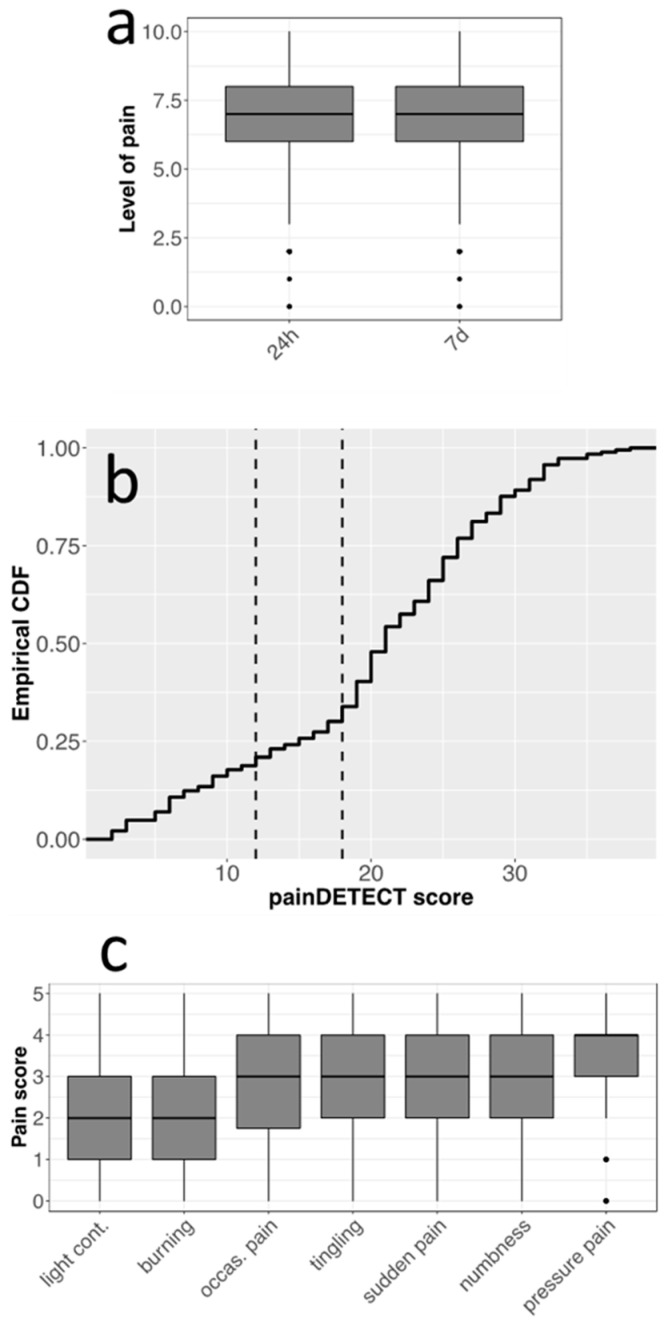
Types of pain and intensities reported by questionnaire respondents (*n* = 186). (**a**) Boxplots of pain intensities (0–10 scale) reported in the last 24 h and 7 days. (**b**) Cumulative frequency of scores in the PD-Q test with cutoffs for pain categories (vertical dashed lines, left: nociceptive pain component; middle: intermediate condition; right: central pain component). CDF: Cumulative Density Function. (**c**) Boxplots of the score distributions (0–5 scale) for the different types of pain reported by patients in the last week.

Participants indicated an average pain intensity level of 6.9 ± 2 on a scale from 0 to 10, referring to the 24 h prior to the questionnaire. This level is very close to that measured by item 1 of FIQR3 (7.2 ± 2.1), referring to one week prior to the questionnaire (Figure 3a), indicating the constant presence of moderate to severe pain. The PD-Q test (Cronbach’s alpha = 0.83) resulted in a significantly unequal distribution of patients among the three pain categories defined by the test score cutoffs, with a marked prevalence of “high” subjects with respect to “low” and “intermediate” ones (Figure 3b). According to the definition followed by PD-Q developers, high scores might correspond to nociplastic pain arising from central pain-processing disorders [26,32]. Moreover, the answers regarding the different types of pain, including pressure pain, numbness, tingling, sudden pain, burning, light contact, and occasional pain, rated on a scale from 1 to 5, showed the highest scores for pressure pain (Figure 3c). The marked prevalence of this pain component is also compatible with a central disorder of pain processing.

The participants were asked to report symptoms other than pain (Table 5). In agreement with the literature data, the vast majority of FM patients experienced fatigue, brain fog, and sleep disturbance. Sensory hypersensitivity was evident in the high frequency of symptoms such as dizziness, visual problems, and migraine. Gastrointestinal issues, including constipation, diarrhea, and nausea, were also highly represented.

Depression, anxiety, and stress are highly related to FM. Their presence and level of severity in the study sample were analyzed by the DASS-21 questionnaire, and the results are reported in Table 6, showing that severe and extremely severe anxiety were prevalent with respect to depression and stress (Cronbach’s alpha = 0.93).

The analysis of responses to the FIQR is reported in Table 7 and Table 8 (Cronbach’s alpha = 0.94). Table 7 shows that 63.9% of the participants scored high in FIQR, indicating a high severity state of the disease. Table 8 shows the descriptive statistics of total FIQR and its three domains related to physical function, overall impact, and symptoms. It should be noted that the Q1 values of all three FIQR domains are equal or higher than the corresponding midpoints of the questionnaire scales (45 for FIQR1, 10 for FIQR2, and 50 for FIQR3). This indicates that for 75% of patients, the physical function was impaired, the overall impact of FM on functional ability and on the perception of reduced function was considerable, and the severity of symptoms ranged from moderately to extremely severe.

### 3.3. Eating Habits and MedDiet

The analysis of the open-ended responses showed that 87% of the participants did not adhere to a specific diet prior to the diagnosis of FM. Moreover, the responses indicated a notable tendency among the subjects to consume high quantities of carbohydrates and simple sugars while simultaneously having a protein intake below the recommended levels. In particular, the excessive consumption of sweets concerned 14% of the subjects, for whom the most preferred products were biscuits, brioches, and chocolate. In addition, breakfast was the most commonly skipped meal, as reported by 8% of patients.

At the time of participation in the survey, meaning long after the FM diagnosis, 68% of the participants rated 7 to 10 on a 0–10 scale measuring the importance of diet for their symptoms. However, only 49% of FM patients declared that they had changed their food habits after the diagnosis and followed specific nutritional advice or dietary patterns. A fraction of 66% of patients decided to consult a physician or nutrition professional to change their diet, while 32% did not. The latter group changed their diet by following the advice of personal trainers or other patients with the same pathology or by seeking information on Internet sites related to FM. A fraction of 2% of patients chose not to answer the question.

The different types of diet followed by the study sample are reported in Table 9. As can be seen, FM patients followed different nutritional advice or dietary patterns at the same time. A dominant trend toward the avoidance of specific food components is evident, particularly with regard to gluten and lactose.

**Figure 4:**
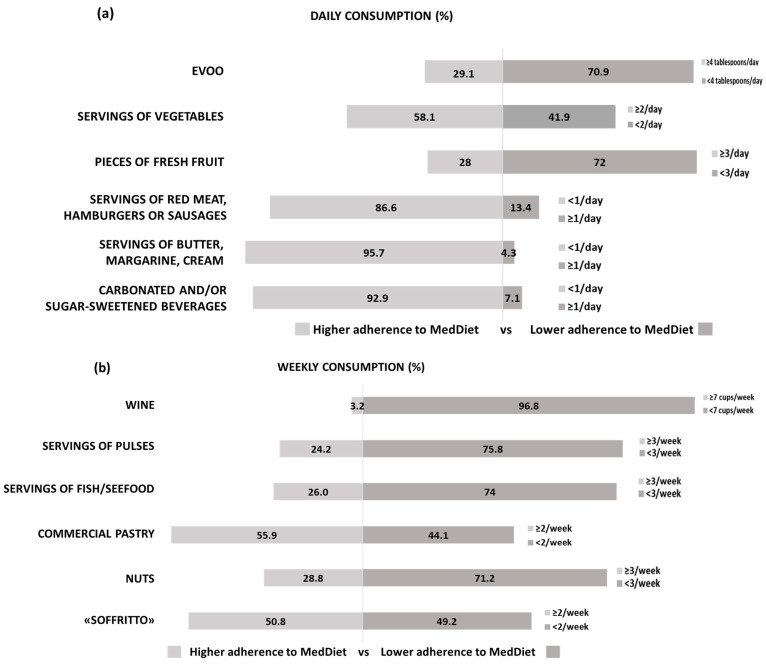
Food consumption out of the total responses received (*n* = 174), assessed on the basis of the PREDIMED-14 items. (**a**) Daily consumption expressed as percentage. (**b**) Weekly consumption expressed as percentage. The light grey bars on the left indicate higher adherence to the MedDiet, while the dark grey bars on the right indicate lower adherence to the MedDiet.

The adherence of the survey respondents to the MedDiet was investigated using the PREDIMED-14 questionnaire. Figure 4 shows the analysis of single items to depict daily (Figure 4a) and weekly (Figure 4b) consumption of food typical of or unrelated to the MedDiet. It is shown that only 29.1% of the participants used extra virgin olive oil (EVOO) daily as the main source of fat in the recommended quantities. The consumption of vegetables and, particularly, fruit need to be improved as well in order to be adherent to the MedDiet in 58.1% and 28% of respondents, respectively. FM patients appeared highly adherent to the MedDiet regarding the daily servings of red meat, fat sources other than EVOO, and sugary drinks (86.6, 95.7, and 92.9% of respondents, respectively) (Figure 4a). Moving to weekly consumption (Figure 4b), conversely to the MedDiet pattern, FM patients drank little wine (possibly because drinking alcohol is not recommended during several pharmacological treatments) and ate pulses, fish, and nuts less than three times a week.

**Figure 5:**
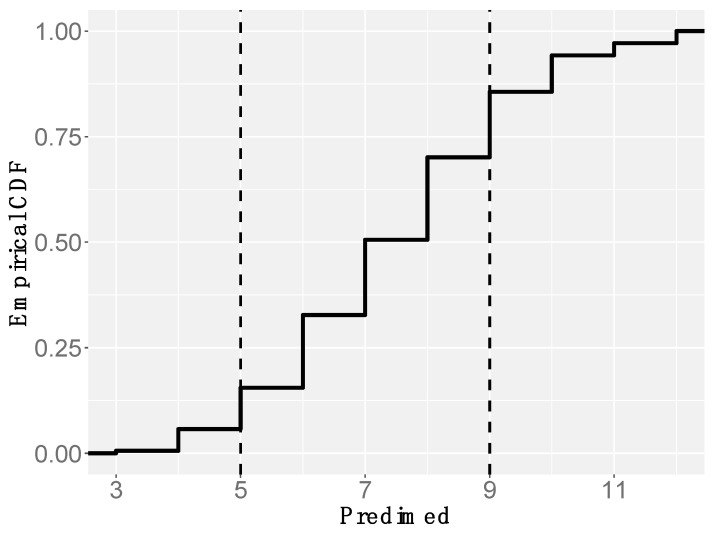
Cumulative frequency of scores in the PREDIMED-14 test with cutoffs for categories of adherence to the MedDiet (vertical dashed lines; left: poor adherence, middle: intermediate adherence; right: good adherence). CDF: Cumulative Density Function (*n* = 174).

The calculation of the PREDIMED-14 score confirmed the above observations and resulted in a significantly unequal distribution of patients among the three adherence categories defined by the cutoffs, with a marked prevalence of intermediate scores (70%) and only 14% of FM patients showing good adherence to the MedDiet (Figure 5).

**Figure 6:**
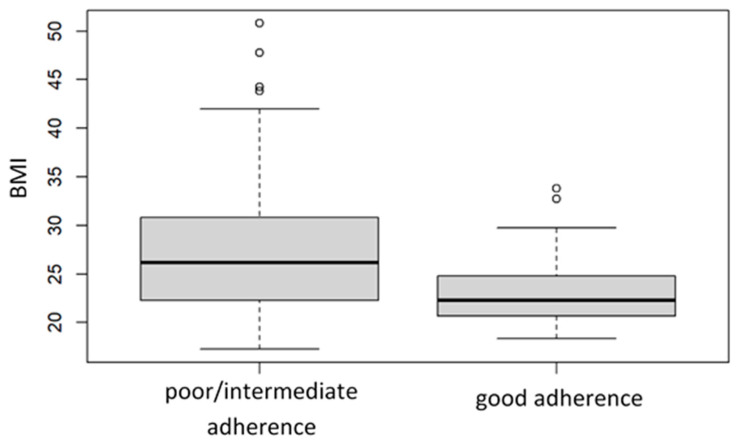
Boxplots of the distributions of BMI values classified on the basis of the level of adherence to the MedDiet according to the PREDIMED-14 cutoffs.

The BMI was negatively correlated with the PREDIMED-14 scores (Figure 6), with a value of Pearson’s correlation coefficient equal to R = −0.28 (*p* < 0.05). Moreover, the BMI was positively correlated with the FIQR and pain intensity values, R = 0.29 and R = 0.14 (*p* < 0.05), respectively.

It is also worth noting that 46% of the participants stated they had a sedentary lifestyle, which is not amenable to a MedDiet program. In addition, only 15% of the study sample reported to be “active” according to the definition of the lifestyle-monitoring program “Sorveglianza PASSI” released by the Istituto Superiore di Sanità, Italian Ministry of Health (https://www.epicentro.iss.it/passi/indicatori/attivit%C3%A0Fisica, accessed on 13 January 2024).

### 3.4. Regression between PREDIMED-14 and FIQR or Pain Metrics

**Figure 7:**
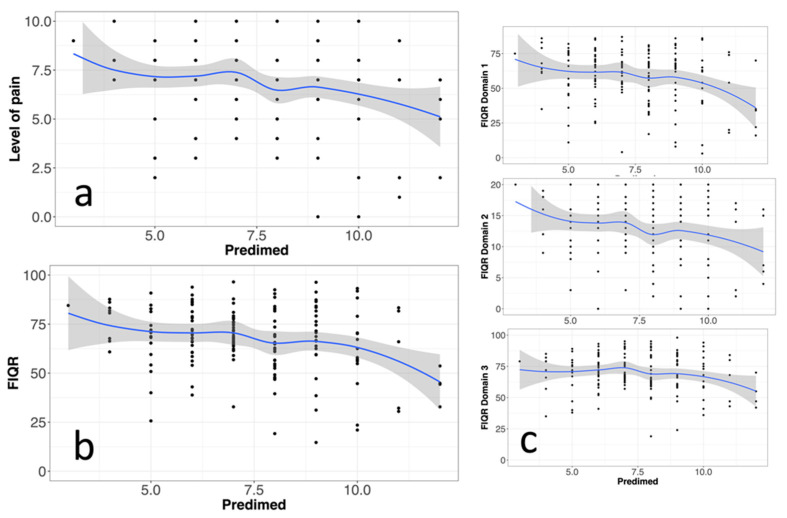
(**a**) Scatter plot of pain intensity scores against PREDIMED-14 scores. (**b**) Scatter plot of total FIQR scores against PREDIMED-14 scores. (**c**) Scatter plot of the scores of each FIQR domain against PREDIMED-14 scores (From top to bottom, domain 1 = physical function (9 items), domain 2 = overall impact (2 items), and domain 3 = symptoms (10 items). The plots show LOESS regression curves by 3 PREDIMED point intervals and their 95% confidence limits (see Table 10 for correlation coefficients and statistical significance).

We first considered the PREDIMED-14 versus the FIQR score and its sub-dimensions and versus the level of pain. A graphical representation of the LOESS regression is plotted in Figure 7, while the statistics for the linear regressions are summarized in Table 10. The PREDIMED-14 scores were positively correlated with FIQR scores and pain intensity, whereas no significant correlation was observed between PREDIMED-14 and PD-Q scores. In the LOESS plots, very similar regression trends between FIQR/PREDIMED-14 and pain intensity/PREDIMED-14 were observed, suggesting that much of the impact of the disease is explained by the pain component of symptoms (Figure 7a,b). In addition, the LOESS plots for each component show that the regression was prevalently accounted for by the physical function and overall impact domains of the FIQR (Figure 7c).

To confirm the previous regression analysis, we used a propensity score weighting technique by considering a dichotomous predictor with two levels: poor/intermediate adherence and good adherence to the MedDiet as derived from the PREDIMED-14 cutoffs. The propensity scores were computed considering the age, education, and lifestyle variables because these variables could presumably exert an influence on the level of adherence to the MedDiet and, therefore, act as confounders in the analysis of the relationship between PREDIMED-14 and FM symptoms. Conversely, we did not consider the BMI because this variable represents a condition that is more likely a consequence of the level of adherence to the MedDiet [33]. The propensity score analysis resulted in significant correlations between FIQR/PREDIMED-14 and pain intensity/PREDIMED-14 (Table 11). This finding suggests that age, education, and lifestyle are not confounders, thus indicating that the observed negative regressions between PREDIMED-14 and either FIQR or pain intensity reflect positive influences of the adherence to the MedDiet on FM symptoms.

Although the use of pharmaceuticals is a highly unbalanced factor (only 13.4% of our sample did not use any of them), we also considered a propensity score analysis by adding this variable as a possible confounder. The significance is still preserved with similar correlations and effect sizes.

## 4. Discussion

### 4.1. Survey Participants Suffered from High-Severity FM

The description of the participants meets the main characteristics of FM patients: middle-aged females, frequently overweight, suffering from chronic moderate-to-severe pain accompanied by a wide variety of symptoms and comorbidities, among which fatigue, sleep problems, and brain fog are the most frequent (Table 1, Table 2 and Table 5) [6,34,35]. The involvement of stress, depression, and particularly anxiety in FM was also confirmed by DASS-21 scores (Table 6). The most prevalent type of pain was pressure pain, which is considered a typical FM diagnostic element [35]. Moreover, 66% of participants reported a high (≥19) PD-Q total score (Figure 3), which is compliant with the concept of nociplastic pain and consistent with the definition of FM as a disorder of central pain processing [26,32].

Due to the complexity of the disease and the lack of biomarkers, the assessment of FM severity is challenging [28,29,36]. Clinicians and researchers frequently rely on questionnaire tools, such as the FIQR, which is particularly useful as an outcome measure in FM clinical trials [37]. The high FIQR scores obtained by most of our survey participants confirmed a high severity level of FM, particularly linked to the impact of symptoms (FIQR3) on physical function (FIQR1) (Table 7 and Table 8).

### 4.2. Survey Participants Extensively Used Pharmaceuticals and Supplements

From the data collected, it is remarkable that the majority of FM patients reported a high level of disease severity despite the usage of a wide spectrum of medications and non-pharmacological therapies. Different pharmaceuticals and supplements are used in an attempt to relieve pain and simultaneously target accompanying symptoms (Table 3 and Table 4 and Figure 2). However, at present, there are only three drugs approved for FM treatment. The FDA approved the anticonvulsant pregabalin and two antidepressants, i.e., duloxetine and milnacipran. Even if there is no gold standard pharmacological treatment for FM, clinical experience suggests starting with antidepressants, followed by anticonvulsants in case of inadequate response [3]. The combined use of these medicaments is confirmed by the correlation matrix of Figure 2. However, in our study sample, the most frequently used pharmaceuticals were anti-inflammatories and analgesics, including ibuprofen, naproxen, ketoprofen, acetylsalicylic acid, paracetamol, tramadol, and corticosteroids. Cyclobenzaprine, a muscle relaxant structurally related to tricyclic antidepressants, recently showed some efficacy against FM pain [38,39] but was rarely used by our survey participants. Relying on their clinical experience, physicians routinely prescribe combined drugs to patients with FM, even if there is no strong evidence to support better outcomes with combination pharmacotherapy as compared with monotherapy [40].

Likewise, current knowledge is too inadequate to recommend a specific use of dietary supplements for the management of FM [19]. Results from our survey revealed that FM patients are very inclined to use supplements and natural remedies, probably thinking that these products are completely harmless compared to drugs. On the contrary, attention should be paid to the excessive and nonspecific use of such compounds, as pharmacological doses of plant extracts and/or isolated active principles can even be toxic or carcinogenic with prolonged usage [41].

In our case histories, the most widely used dietary supplement was vitamin D. Some evidence supports the benefit of vitamin D supplementation in the treatment of FM, as vitamin D levels are significantly lower in FM sufferers than in healthy controls [42,43]. It is believed that vitamin D supplementation can modulate pain in several syndromes characterized by chronic widespread pain, including FM [44]. A deficiency of vitamin D impacts the nociceptive innervation of skeletal muscle, leading to increased innervation and heightened sensitivity to musculoskeletal pain [45]. Dietary vitamin D sources are limited and include fish, egg yolk, and offal, which may not be routinely consumed in different countries or in vegetarian/vegan diets [46]. Given that, for many people, even sun exposure is often insufficient, it can be concluded that vitamin D supplementation is desirable for patients with FM, particularly when considering that most of these patients are post-menopausal women.

Our data indicate that magnesium is the second most commonly used dietary supplement among FM patients. Other studies corroborate this, highlighting magnesium, after multivitamins, as one of the most frequently employed supplements in the attempt to manage FM symptoms [47]. In addition, there is evidence indicating that FM patients often have a magnesium-deficient diet associated with classic FM symptoms, such as muscle pain, fatigue, sleep difficulties, and anxiety [48]. A particular model of a Mediterranean Diet enriched in magnesium and tryptophan was administered to a population of female FM patients, showing beneficial psychological effects on emotional processing, traditionally impaired by this disease. Specifically, a reduction in anxiety, mood disorders, fatigue, and depression, as well as dissatisfaction with body image, was observed [49]. Some authors suggested an analgesic function of magnesium. In the context of FM patients, Andretta et al. found an inverse correlation between dietary intake of magnesium and calcium and the number of tender points and a direct association with pain threshold. At the same time, a low assumption of these two minerals was associated with worsening pain [50]. Magnesium blocks N-methyl-D-aspartate (NMDA) receptors in a voltage-dependent manner, thus playing a role in preventing central sensitization. Indeed, when the NMDA receptor is inhibited, central sensitization and pain hypersensitivity decrease. In contrast, glutamate, substance P, and calcitonin gene-related peptide (CGRP) act in the opposite direction to magnesium, inducing an activation of NMDA channels [43]. Substance P is known to be a neurotransmitter involved in pain perception. The increase in substance P correlates with magnesium deficiency and increased pain intensity in FM disease [51].

Beyond pain, a significant proportion of FM symptoms also relates to the gastrointestinal tract, supporting the hypothesis of a close association between FM and irritable bowel syndrome (IBS) [52]. Moreover, evidence shows that the gut microbiota of FM patients is altered in its composition, suggesting a role of dysbiosis in the pathogenesis of the disease [9,53,54]. In our survey, constipation, colitis, or diarrhea were reported by 48% and 45% of patients, respectively (Table 5). However, only 25.8% of the participants declared the use of prebiotics and/or probiotics. Despite the existence of a rationale for targeting the gut microbiota in the treatment of IBS, the grade of recommendation for probiotic use is still low due to the lack of high-quality studies [55]. The same is true when considering the efficacy of gut microbiota modulation in alleviating more typical FM symptoms, including pain [19]. However, the advent of well-designed research in this field is warranted, given the growing interest in the role of the microbiota–gut–brain axis in health and disease [56]. An alteration in the gut–brain axis was linked to the emergence of different types of pain, while the gut microbiota appears to play a crucial role in the central sensitization of chronic pain by influencing microglia, astrocytes, and immune cells. Additionally, disruptions in the gut microenvironment may contribute to chronic visceral pain [14]. The disequilibrium of microbial populations resident in the gut, consisting of dysbiosis, is associated with the improper activity of the gut–brain axis and an alteration of the centrally processing of sensory input [57]. In fact, while acute changes in this interoceptive feedback can lead to temporary functional brain modifications (as seen in gastrointestinal infections), persistent alterations are linked to neuroplastic changes in the brain, which could trigger nociplastic pain [58]. A comprehensive understanding of the gut microbiome’s impact on the pathophysiology of pain opens avenues for the development of analgesic therapies focusing on microorganisms as beneficial approaches to alleviating pain [57].

### 4.3. FM Patients Should Improve Lifestyle and Eating Habits to Improve Wellbeing

The only ‘strong’ recommendation on FM management released by EULAR (European League Against Rheumatism) is in favor of physical exercise [13]. Our data show that FM patients do not seem to observe an active lifestyle but rather a sedentary lifestyle, which is the opposite of the Mediterranean style [59]. A large fraction of the study sample described their lifestyle as sedentary (46%). Only 15% of respondents defined themselves as active, practicing moderate physical activity for at least 30 min for 5 days/weekly and/or intense physical activity for more than 20 min 3 times a week. A fraction of 39% of participants reported maintaining a moderately active lifestyle, indicating that during their free time, they engage in physical activities two to three times a week, such as walking, cycling, swimming, and gymnastics. Based on the available literature, patients with FM show sub-optimal physical performance and marked functional disability caused by exercise intolerance, which is closely linked to fatigue and pain [60]. For instance, a pilot case–control study showed that female FM patients with overweight/obesity had lower levels of activity both at home and at work when compared to healthy females. Normal-weight women with FM showed reduced scores of physical activities at work and home, too [61]. Yet, according to a recent study, low-intensity physical exercise, including a program of resistance and coordination, improved psychological condition, pain perception, quality of life, and overall physical conditioning in FM patients [62].

Physical exercise can also help FM patients maintain a healthy weight range. The descriptive characterizations of the participants in our survey confirmed the strong association between FM and overweight or obesity, already underpinned by previous studies [60,63]. Despite this evident association, the link between obesity and FM has not yet been clearly delineated, so several hypotheses were formulated in this regard. It is hypothesized that obesity may play a role as a potential risk factor for the development of FM or also represent an enhancing element for symptomatology [64]. For example, obesity could contribute to the inflammatory environment that amplifies and prolongs nociceptive signals originating from the periphery [60]. Kadayifci et al. [65] also suggested weight management, in combination with daily physical activity, as a fundamental part of therapy for FM, as well as to improve quality of life and decrease excessive weight. In fact, in FM, a higher BMI correlates with decreased quality of life, sleep, and physical performance. At the same time, obesity is associated with an increased risk of depression [66,67]. Accordingly, in our study sample, the BMI was positively correlated with pain intensity and FIQR scores.

Overweight and obesity, as well as depression and sleep problems, can be related to eating disorders in FM. For example, binge eating can be a way to cope with the emotional distress caused by FM symptoms [68]. The nucleus accumbens and the ventral tegmental area have been implicated in binge eating disorder and obesity [69]; these brain regions are not directly involved in pain processing, and we could not find published data discussing a possible overlap of brain areas involved in pain and binge eating. Indeed, an interplay between these two aspects cannot be excluded, but a detailed analysis is beyond the scope of this paper.

A personalized balanced nutritional plan represents the best way to ensure the adequate intake of macro- and micro-nutrients as well as microbiota–gut–brain axis signaling. Thus, besides an active lifestyle and appropriate pharmacological and non-pharmacological therapies, diet represents a promising, powerful approach to alleviating FM symptoms [70]. According to one of our previous questionnaire studies, dietary interventions account for the highest patient satisfaction among non-pharmacological therapies [6]. This is in line with our present results, showing that 68% of participants believed that diet was of considerable importance in controlling their symptoms, giving it a score of 7 to 10 on a 0–10 scale. Confirming this is the fact that almost half of the subjects decided to change their diet after the diagnosis. Previous studies demonstrated that once patients are diagnosed, they often change food habits in an attempt to alleviate FM symptoms, sometimes seeking the help of health professionals to implement their diet [71,72]. Our results showed that 66% of FM patients decided to follow a physician or nutrition professional advice. On the other hand, 32% of the sample opted to rely on other professional figures, such as personal trainers, or to follow information found on the internet or from fellow patients with the same condition. Before the diagnosis of FM, participants did not adhere to a specific dietary pattern. It was common to find an unbalanced diet among respondents, indicating a trend in the overconsumption of carbohydrates and simple sugars, together with scarce protein consumption. After the diagnosis, several dietary approaches are followed by people with FM, but there is currently no evidence as to which are most helpful in treating the condition [3,73,74].

Among the subjects who participated in our study, a diverse range of adopted diets emerged (Table 9). The lactose-free diet was predominant for about 65% of the answers collected, followed by a gluten-free diet, 58%; a Mediterranean-based diet, 32%; vegetarianism, 14%; a low Fermentable Oligosaccharides, Disaccharides, Monosaccharides, and Polyols (FODMAP) diet, 11%; and ketogenic diets, 10%. According to a recent systematic review, elimination diets, such as gluten-free diets, led to inconclusive results regarding their effectiveness in controlling FM symptoms. This may be due to the fact that such dietary patterns do not emphasize the consumption of plant-based foods, such as fruit, vegetables, and nuts, nor do they provide a clear guideline for reducing the intake of processed foods [10]. Moreover, such dietary schemes, with the exception of the ketogenic diet, would not be aimed at weight loss, which, as aforementioned, is considered useful in FM patients [75]. Pagliai et al. [19] claimed that dietary patterns rich in fiber, anti-oxidants, and plant-based foods, such as the MedDiet diet, appear to be effective in reducing FM symptoms. Specifically, diets rich in plant food appear to reduce anxiety, depressive states, and chronic pain; improve cognitive and gastrointestinal functions; as well as enhance sleep quality in individuals affected by this condition [76].

### 4.4. FM Patients Should Increase the Adherence to the MedDiet

The MedDiet is considered a plant-based dietary pattern, known for its positive effects in combating inflammatory states and oxidative stress inflammation but also cognitive decline, neurodegeneration, and the development of tumors [77]. Specifically, in the Mediterranean model, there is a notable emphasis on whole grains and plant-based foods, including pulses, vegetables, fresh fruits, olives, nuts, and seeds. Lean meats such as fish and poultry are also prioritized. Daily but moderate consumption of low-fat dairy is recommended, while the intake of red and processed meats is considerably restricted. EVOO is the principal source of dietary fats and, concurrently, a pivotal component contributing to its benefits, primarily attributable to phenolic compounds such as oleocanthal, oleuropein, and tyrosol. Water and herbal teas are necessary to meet hydration needs, while prudent consumption of wine is recommended in accordance with social mores. In addition to the dietary components, the basis of the Mediterranean style includes daily moderate physical activity of at least 30 min [78,79].

The results reported in Figure 4 and Figure 5 show some critical issues on the part of FM patients in following MedDiet recommendations about the consumption of different foods, leading to poor/intermediate adherence to this dietary style. Our data are in line with those of a recent survey measuring a low–medium adherence to the MedDiet in a sample of Italian adults [80]. Thus, it seems that the condition of FM patients does not modify the frequency of adhesion to MedDiet recorded in the whole population. 

Of significance, we found a negative correlation between adherence to the MedDiet and BMI value, intensity of pain, and the FIQR scores (Figure 6 and Figure 7 and Table 10 and Table 11), thus evincing a beneficial effect of the MedDiet on the overall FM impact. The lack of knowledge on FM pathogenesis makes it difficult to speculate about a mechanism underlying such a beneficial effect, and several hypotheses remain open, including the involvement of the microbiota–gut–brain axis, as discussed above, but also the anti-oxidant and anti-inflammatory pathways. As highlighted by a recent systematic review, individuals who prefer a diet based on plant products and whole grains in the Mediterranean style exhibit a negative correlation with inflammatory markers. Conversely, those following a Western-type diet, characterized by a high intake of simple sugars and hydrogenated fats, show a positive association with the markers of systemic inflammation. In particular, the MedDiet proved effective in reducing IL-6 and CRP in individuals with metabolic syndrome compared to controls [74]. O’Mahony et al., in their recent meta-analysis, observed statistically significant differences in the cytokine profile between healthy and FM individuals. FM patients are characterized by a heightened proinflammatory component, particularly in the levels of IL-6, IL-8, and TNF. Additionally, there is the presence of IL-10, which exerts an anti-inflammatory action, along with chemokines [81]. Therefore, it is believed that further studies are necessary to define the role of cytokines in the pathology and the role of diet as well.

Regarding psychological aspects, it was reported that in both healthy adults [82] and in women with FM [83], consuming the optimal amount of fruit and vegetables is associated with better mental health, whereas excessive consumption of meat products is related to higher levels of depression. Our results failed to demonstrate a correlation between adherence to the MedDiet and psychological well-being, particularly depression, anxiety, and stress, as measured by the DASS-21 questionnaire. However, in our opinion, this result strengthens the significance of the beneficial effect of the MedDiet against pain, as it rules out the possibility of a nonspecific, placebo-like effect of the diet. This is further acknowledged by propensity score analysis (Table 11), which points to positive influences of the MedDiet on FM pain and physical/functional impact. Moreover, the lack of correlation between the PREDIMED-14 and PD-Q scores suggests that the MedDiet can help decrease FM pain intensity without affecting the type of pain, in line with the concept of FM as a central disorder of pain processing. In this view, the possibility that the MedDiet could play a central role in a complete FM remission appears unlikely. Nevertheless, our results suggest the beneficial effects of the MedDiet in mediating FM severity. When framed inside a multimodal therapeutic approach, the MedDiet and appropriate supplementation with bioactive food components could help reduce the intake of multiple types of drugs that, according to clinical evidence and patients’ satisfaction, bring more unwanted side effects than beneficial outcomes in most cases [3,6].

It is worth reiterating that a multi-disciplinary therapeutic approach that starts from non-pharmacological strategies and assures the balance between the benefits and the risks of treatments is a primary recommendation of EULAR (European League Against Rheumatism) for FM [13]. In this regard, a practical implication of the results of our questionnaire is that such multi-disciplinary patient care cannot disregard nutritional assessment and intervention performed by a dietician or nutrition expert. Various dietary approaches are followed by people with FM, many of which involve the elimination of food groups, such as vegan, vegetarian, or lactose-free diets. Following a dietary approach without the help of a specialist may result in the unwarranted elimination of nutrients that are essential for health and in unjustified dietary sacrifices. Moreover, people who cannot change their diet (e.g., for health or economic reasons) can still benefit from expert advice, with particular emphasis on the intake of anti-oxidants and personalized nutritional supplementation.

### 4.5. Limitations of the Study

Like all anonymous online surveys, this study has intrinsic limitations that cannot be avoided, including the self-selection of participants (more prone to/capable of/interested in responding), non-probability snowball sampling effects due to uncontrollable sharing of the survey link through social networks, and untruthful answers to self-reported questions. The use of multiple pharmaceuticals and supplements by FM patients could influence the severity of pain. Further uncertainty is linked to individualized responses related to drug metabolism, receptor sensitivity, interactions with other drugs, etc.

Nevertheless, our study sample completely meets the typical FM features, such as gender bias, pain characteristics, the prevalence of symptoms, and comorbidities, thus confirming the validity of our data. Also, our data showed that the use of pharmaceuticals was not a confounder in the propensity score analysis.

## 5. Conclusions

The data from our survey highlight the need for educational programs tailored for FM patients in terms of healthy lifestyles, physical activity, diet, and the appropriate use of dietary supplements when evidence of some efficacy exists for such supplementation. This is in line with EULAR recommendations suggesting that the first-line management of FM patients should focus on patient education and non-pharmacological therapies [13]. Diet therapies should be tailored to the specific needs of the individual, taking into account the absence or presence of comorbid obesity, gastrointestinal disturbances, and micronutrient deficiency. Our findings suggest that a classic MedDiet, with possible personalized modifications and supplements, can improve FM symptoms, but high-quality intervention studies are needed to evaluate the therapeutic potential of different dietary patterns and supplementation in FM.

## Figures and Tables

**Table 1:** Percent distribution of demographics in FM participants (*n* = 186).

Sex	Female	Male	No Answer			
**FM**	96.7	2.7	0.5			
**Education**	Primary	Lower secondary	Upper secondary	Academic degree	PhD or equivalent	
	0.5	25.4	51.9	12.9	9.2	
**Marital status**	Single	Married/cohabitant	Separated/divorced	Widowed		
	15.0	61.3	20.9	2.7		
**Employment**	Grey-collar	White-collar	Blue- collar	Freelance	Unemployed/retired	No answer
	40.3	1.6	6.0	8.0	41.4	2.7

**Table 2:** Descriptive statistics of clinical features in FM participants (*n* = 186).

	Q1 ^a^	Median	Mean ± SD	Q3 ^b^
Patient age (years)	48.5	56	54 ± 10	60.5
Height (cm)	158	160	162 ± 6.8	166
Weight (Kg)	58	67	69.4 ± 16.2	79
BMI	21.8	25.6	26.5 ± 6.2	30.1
Disease duration (years)	5	9	10.7 ± 7.7	13

^a^ first quartile; ^b^ third quartile.

**Table 3:** Use of pharmaceuticals (a) and of supplements (b) by FM patients (*n* = 186).

(a)	Pharmaceuticals	Frequency (%)	(b)	Supplements	Frequency (%)
	Anti-inflammatories/ analgesics	60.8		Vitamin D	65
	Antidepressants	40.3		Magnesium	49.5
	Antineuralgics/anxiolytics	33.9		Prebiotics/probiotics	25.8
	Cannabinoids	20.4		Melatonin	21
	Corticosteroids	14		Anti-inflammatories	21
	None	13.4		Anxiolytics	21
				Anti-oxidants	19.9
				Preparations for joints	18.3
				Immunostimulants	17.2
				Multivitamin/Multimineral	17.2
				Turmeric	13
				None	14

Frequencies ≤ 10% are not reported.

**Table 4:** Use of non-pharmacological mind–body therapies by FM patients (*n* = 186).

Therapy	Frequency (%)
Physiotherapy	20
Meditation	20
Relaxation techniques	14.5
Yoga	14
Massage	12.4
Acupuncture	8.6
None	42.5

**Table 5:** Percent frequency of non-pain symptoms reported by FM patients (*n* = 186).

Symptom	Frequency (%)
Fatigue	84.4
Brain fog	80.1
Sleep disturbance	78.5
Dizziness	66.7
Anxiety	59.1
Photophobia	51.1
Migraine	49.5
Constipation	48.4
Colitis/diarrhea	45.2
Depression	37.1
Diplopia	34.4
Nausea	27.4

Frequencies ≤ 10% are not reported.

**Table 6:** Percent frequencies of the severity levels of depression, anxiety, and stress in the study sample, as defined by the DASS-21 cut-off points (*n* = 170 for Depression, *n* = 179 for Anxiety, *n* = 182 for Stress).

Severity	Depression	Anxiety	Stress
Normal	24.4	14	35.9
Mild	14.3	10.1	14.4
Moderate	28.6	29.8	25.4
Severe	9.5	12.4	17.7
Extremely severe	23.2	33.7	6.6

**Table 7:** Frequency distribution of the FM severity states in the study sample as categorized by the FIQR cut-off points (*n* = 169).

FM Severity State	Frequency (%)
Remission	3
Mild	8.3
Moderate	24.9
High	63.9

**Table 8:** Descriptive statistics of the FIQR and its 3 domains.

FIQR Scores	Min	Q1 ^a^	Median	Mean ± SD	Q3 ^b^	Max
Total	14.7	59.3	69.3	68.0 ± 17.1	81.3	100
FIQR1 (Physical function)	3.0	49.0	64.5	59.3 ± 19.2	74.0	90
FIQR2 (Overall impact)	0	10.0	14.0	13.1 ± 5.1	17.0	20
FIQR3 (Symptoms)	19.0	62.0	71.0	70.4 ± 14.8	82.0	100

^a^ first quartile; ^b^ third quartile.

**Table 9:** Frequency distribution of the types of diet followed by FM patients (*n* = 91).

Type of Diet	Frequency (%)
Lactose-free	64.8
Gluten-free	58.2
Low-calorie Mediterranean	31.9
Vegetarian	14.3
Low FODMAP	11
Ketogenic/ VLCKD (800 kcal)	9.9
Vegan	5.5
Other/personalized	36.3

FODMAP: Fermentable Oligosaccharides, Disaccharides, Monosaccharides and Polyols; VLCKD: Very Low-Calorie Ketogenic Diet.

**Table 10:** Summary statistics of the regression between PREDIMED-14 and FIQR or pain metrics.

	FIQR/ PREDIMED-14	Pain Intensity/ PREDIMED-14	PD-Q/ PREDIMED-14
R ^a^	0.238	0.20	0.055
Slope ^b^	–2.12	–0.21	–0.24
p	0.003	0.008	0.475

^a^ Pearson’s correlation coefficient; ^b^ average variation of the metric for one-unit increase in PREDIMED-14.

**Table 11:** Summary statistics of the regression between dichotomous PREDIMED-14 and FIQR or pain metrics.

		FIQR/ PREDIMED-14	Pain intensity/ PREDIMED-14
	R ^a^	0.222	0.202
Without propensity score	Delta ^b^	–10.54	–1.17
	p	0.0005	0.0084
	R ^a^	0.183	0.235
With propensity score	Delta ^b^	–6.929	–1.09
	p	0.0216	0.0018

^a^ Pearson’s correlation coefficient; ^b^ difference between the means of the metric, divided into two groups as classified by the PREDIMED-14 conditions, poor/intermediate adherence, and good adherence. Propensity score analysis was conducted considering the age, education, and lifestyle variables. The analysis was not performed on PD-Q/PREDIMED-14 due to nonsignificant correlation (see Table 10).

## Data Availability

For ethical reasons the datasets used and analyzed during the current study are available from the corresponding author upon reasonable request.
